# Effects of digital physical activity interventions on muscle mechanical function in community-dwelling older adults: a systematic review and meta-analysis

**DOI:** 10.1186/s11556-025-00380-z

**Published:** 2025-09-02

**Authors:** Carlo della Valle, Charlotte Gatti, Alessio Bricca, Valentina Mancini, Orgesa Qipo, Nerijus Masiulis, Jon André Christensen, Mohammad Mosaferi Ziaaldini, Günay Yildizer, Soran Aminiaghdam, Paolo Caserotti

**Affiliations:** 1 University of Verona, Verona, Italy; 2 University of Molise, Campobasso, Italy; 3 University of Vienna, Centre for Sport Science and University Sports, Vienna, Austria; 4 University of Southern Denmark, Odense, Denmark; 5 Foro Italico University of Rome, Rome, Italy; 6 Vrije Universiteit Brussel, Brussels, Belgium; 7 Lithuanian Sports University, Kaunas, Lithuania; 8 Næstved, Slagelse and Ringsted Hospitals, Næstved, Denmark; 9 Ferdowsi University of Mashhad, Mashhad, Islamic Republic of Iran; 10 Eskisehir Technical University, Eskişehir, Turkey; 11 UMIT Tirol– Private University for Health Sciences, Medical Informatics and Technology, Innsbruck, Austria

**Keywords:** Older adults, Digital exercise, Muscle mechanical function

## Abstract

**Aim:**

To investigate the effect of digital exercise interventions on muscle mechanical function in community-dwelling older adults aged 60 and above.

**Methods:**

Systematic review of randomised controlled trials (RCTs) retrieved from PubMed, EMBASE (Ovid), the Cochrane Central Register of Controlled Trials, and Web of Science until end of March 2024. The Cochrane RoB2.0 tool and GRADE were employed for quality assessment. We performed meta-analysis using random-effects model and sub-group and meta-regression analyses to investigate the robustness of the findings. GRADE was used to assess the overall certainty of the evidence.

**Results:**

Thirty RCTs, comprising 1697 participants with a mean age of 71.27 years, were included in the data analysis. The meta-analysis revealed a significant overall effect of technology-based physical activity intervention on muscle mechanical function (Hedge’s g = 0.27, p = < 0.001). In the sub-analysis, 18 studies focused on interactive interventions on handgrip strength (SMD 0.10, 95% CI -0.17 to 0.38) and leg strength (SMD 0.56, 95% CI 0.19 to 0.93). The overall certainty of the evidence was deemed low.

**Conclusion:**

Digital interventions focusing on physical activity interventions have generally shown small but significant improvements in older adults. Interactive and semi-interactive interventions were effective, while passive ones were not.

**Supplementary Information:**

The online version contains supplementary material available at 10.1186/s11556-025-00380-z.

## Introduction

Ageing is associated with structural and functional changes in the neuromuscular system, including a progressive loss of muscle mass, a decrease in muscle density, an increase in fat infiltration into muscle tissue, and reduced voluntary activation [33, 40]. These lead to significant deterioration in muscle mechanical function (MMF), including reduced muscle strength, muscle power, explosive force and muscle quality (i.e., force per unit of muscle mass) [14]. These changes generally begin around the fourth/fifth decade of life and accelerate thereafter [2, 26]. Importantly, several studies suggest that these changes are associated with a higher likelihood of falls in older adults [36], poor physical function, and an increased risk of functional dependency [13].

Exercise is a cornerstone in counteracting the neuromuscular decline associated with the ageing process. Several systematic reviews have demonstrated that it can enhance neuromuscular quality, muscle density and strength [1, 5, 8, 10, 21, 32, 42].

However, the effect of digital interventions on muscle mechanical function (e.g., muscle strength and power) in older adults has only been investigated by few studies with contrasting results both on muscle function and functional capacity [12, 16, 18, 25]. This discrepancy in results highlights the lack of a systematic review integrating and summarising the individual studies.

The variation in results from randomized controlled trials may be attributed to differences in the characteristics of digital interventions (e.g., frequency, intensity, volume, progression) and mode of delivery (e.g., passive versus interactive digital interventions, supervised versus non-supervised interventions).

Therefore, the purpose of this systematic review was to investigate the effects of digital exercise interventions on muscle mechanical function in older community-dwelling.

### Methods

The present systematic review and meta-analysis was conducted in accordance with the Cochrane Handbook [19] and reported following the Preferred Reporting Items for Systematic Reviews and Meta-analyses (PRISMA) guidelines [29]. The protocol was registered in PROSPERO (CRD42024535437) on May 3, 2024, before performing the literature search.

### Eligibility criteria

The eligibility criteria, outlined below in Table 1, follow the PICOS criteria, encompassing population, intervention, comparator, outcome, and study design.

**Table 1:** Eligibility criteria

	Inclusion criteria	Exclusion Criteria
**Participants**	Community-dwelling older adults aged 60 years and over, as defined by the included trials. Age: >60 years old Mean age: 70 years old Human subjects Defined as healthy, despites age-related impairments and functional limitations Studies may include multimorbid community-dwelling adults, as long as the study does not focus exclusively on any disease specific population (e.g., diabetes, COPD, arthritis). *Examples*: Osteoarthritis/porosis Reduced muscle mass Hearing loss Vision impairments Cognitive decline Balance and coordination issues Reduced mobility Decreased lung function Urinary incontinence	Age: ≤60 years old Non-human subjects Institutionalized older adults or with a specific clinical chronic condition not related to ageing. Studies targeting disease-specific older adults, including those with frailty. *Examples*: Chronic obstructive pulmonary disease (COPD) Rheumatoid arthritis Autoimmune disorders Traumatic brain injury (TBI)
**Type Intervention**	Digital physical activity intervention: *Examples*: Strength training Resistance training Power training E-health M-health Interventions based on more than a single bout of exercise.	Interventions limited to a single bout of exercise. Studying acute effects (during or < 24 h after intervention)
**Publication status**	Published	N/A
**Publication language**	English	N/A
**Comparison**	Control groups receiving usual care, pharmacological and non-pharmacological intervention (e.g., diet), educational materials during the intervention, or balance/yoga intervention.	No control groups Control groups participating in physical activity.
**Outcomes**	Exercise-induced changes in Muscle mechanical function: *Example*: Muscle strength, Muscle force, Muscle power, Explosive muscle force, Rate of force development, Force fluctuation, Impulse, Force-velocity profile	No outcomes for muscle mechanical function reported.
**Study design**	Randomised controlled trials Cross-over RCTs	• Feasibility studies, case-control design, cross-over design, case report, case series, review papers, guidelines, article synopsis, pre-registrations, protocol papers, unpublished work, conference abstracts, or expert opinions.

### Information sources

RCTs were searched in the PubMed, EMBASE (via Ovid), Cochrane Central Register of Controlled Trials and Web of Sciences from inception until 31 March 2024. We also screened the references of the included articles and grey literature to identify eligible RCTs, in line with the Cochrane handbook recommendations.

### Search strategy

A search matrix was developed in collaboration with international researchers from PhysAgeNet (Network on Evidence-Based Physical Activity in Old Age), COST (European Cooperation in Science and Technology) Action CA20104 (*PhysAgeNet*,* Cost-Action CA20104*, 2022), adhering to the PICOS criteria (Table 1). Preliminary searches on PubMed were conducted to refine the matrix. The complete search matrix is available in the Supplementary Material Table 2.

Medical Subject Headings (MeSH) as well as Text Word (TW) terms and keywords pertaining to PICOS were included. The terms within the categories of participants (e.g., “Aged” [MeSH] OR “Older Adult*”), intervention (e.g., “Exercise” [MeSH] OR “Physical Activity”) and outcome (e.g., “Muscle Strength” [MeSH] OR “Muscle Mechanical Function”) were combined with the Boolean operator “AND”. The three reviewers conducted the searches (CG, VM, OQ). The PubMed search was filtered according to the Cochrane RCT filter [27], with additional keywords added. The Embase and Web of Science databases were searched using the RCT filter.

### Data collection

The modified version of Cochrane Collaboration data collection form for intervention reviews (RCTs only) [20] was used. When studies involved two different digital interventions (group A and B) and one control comparator group (C), the comparator group was divided into two smaller sample size groups. Groups A and B were compared to group C, and the results were reported as two separate study comparisons. This procedure is in accordance with the Cochrane handbook [20].

For continuous outcomes (mean and standard deviation, standard error or 95% Confidence Interval), we extracted the following data:

- Trial characteristics: location of the trial (e.g., Country), number of participants allocated to the digital intervention and comparator groups, respectively.
- Participant characteristics: age, % female, body mass index (BMI).
- Intervention and comparator characteristics: type of the digital intervention/comparator interventions, format of intervention (interactive interventions, where participants exercise with consoles (e.g., Nintendo Wii^®^ or Microsoft Xbox Kinect^®^) or devices and actively interacted with the device (e.g., responding to visual stimuli through movements recorded by the camera or a remote controller with sensors); semi-interactive interventions that use video, application and device-based techniques (e.g., a visual stimulus is provided, but the device does not record and respond to the participant’s action); passive interventions that use devices or telephone calls, where there is no sensory cue or possibility for interaction with the device); frequency of the sessions (times per week), intensity of the session (e.g. % of perceived intensity), duration of the interventions (weeks), presence of supervision (yes, no or a combination), adherence to the intervention (number of sessions attended out of the total number of planned sessions), and primary types of exercise (e.g., neuromuscular, aerobic).
- Outcome characteristics: magnitude of measured changes (e.g., change in muscle strength), anatomical site of the assessment (e.g., upper body, lower body), type of muscle mechanical function (e.g., muscle strength, muscle power, explosive muscle force, rate of force development).

### Study risk of bias assessment

We assessed the risk of bias with Cochrane Rob tool 2.0 across five different domains: randomisation process, deviations from the interventions, missing outcome data and measurement and selection of the reported results) [37]. Theis assessment was conducted by three independent reviewers (CG, VM, OQ). The fourth independent reviewer (PC) resolved disagreement on the judgments through discussion in line with the Cochrane handbook guidance. The reviewers answered one or more signalling questions within each risk of bias domain and these answers lead to judgments of “low risk of bias,” “some concerns,” or “high risk of bias”. The decisions within each domain resulted in an overall risk-of-bias judgment for the assessed outcome.

### Certainty assessment

Two independent reviewers (AL and JAC) used the *Grading of Recommendations Assessment*,* Development and Evaluation* (GRADE Working Group Tool)to assess the certainty of evidence for the main outcome of MMF, separated into handgrip strength (HS) and leg strength (LS). The range of certainty spans from from very low *(“any estimate of effect is very uncertain”)* to high *(“future research is very unlikely to change the estimates of effect”)*. The domains assessed include: (i) study limitations (risk of bias), (ii) inconsistency (across studies, e.g., heterogeneity), (iii) indirectness (whether evidence answers the research question, e.g., poor generalisability of the findings for the population defined in the PICO) and (iv) imprecision (e.g., width of the 95% CI) [35].

### Effect measures

We conducted a meta-analysis to assess the effect of the interventions on MMF, using the standardised mean difference (SMD), with 95% confidence interval (CI), adjusting for the Hedge’s g to account for potential bias due to the small sample size. THE SMD difference was estimated as the difference between the mean score of the intervention and control groups, divided by the pooled standard deviation SD of the final score. The effect size was interpreted as small effect (≤ 0.2), a moderate effect (0.5), or a significant effect (≥ 0.8) following the guidelines by Hedge’s g Statistic (2018) [6, 27].

### Statistical analysis

Analyses were conducted using the statistical program STATA (STATA/BE 18.0) with the package ‘meta esize’. To investigate statistical heterogeneity, subgroups analysis were performed on the outcome domains (handgrip strength (HS) and leg strength (LS)), and intervention characteristics (digital component (DC), type of intervention (ToI), supervision, exercise intensity (rate perceived exertion (RPE) Borg scale (6–20)). Meta-analyses were presented as forest plots, and random effects models were used to estimate relative risks with 95% CI. Relevant study-level covariates were defined as those that can decrease inconsistency, as measured by the I² statistic (and thus the between-study variance). The I² was used to report statistical heterogeneity (Higgins and Thompson, 2002) and was interpreted on a continuous scale ranging from 0 to 100%, with 100%indicating maximal statistical heterogeneity. Meta-regression analyses were performed to investigate the impact of study-level participant baseline characteristics, as pre-specified (age, female (%), body mass index (BMI), disability severity (timed up-and-go (TUG) test), attendance rate (the proportion of sessions attended out the total number of sessions), total number of sessions, and session duration (minutes)). Bubble plots were conducted to visually represent the relationship between study-level covariates and outcome alongside regression coefficients and 95%CIs.

## Results

### Study selection and characteristics

**Fig. 1:**
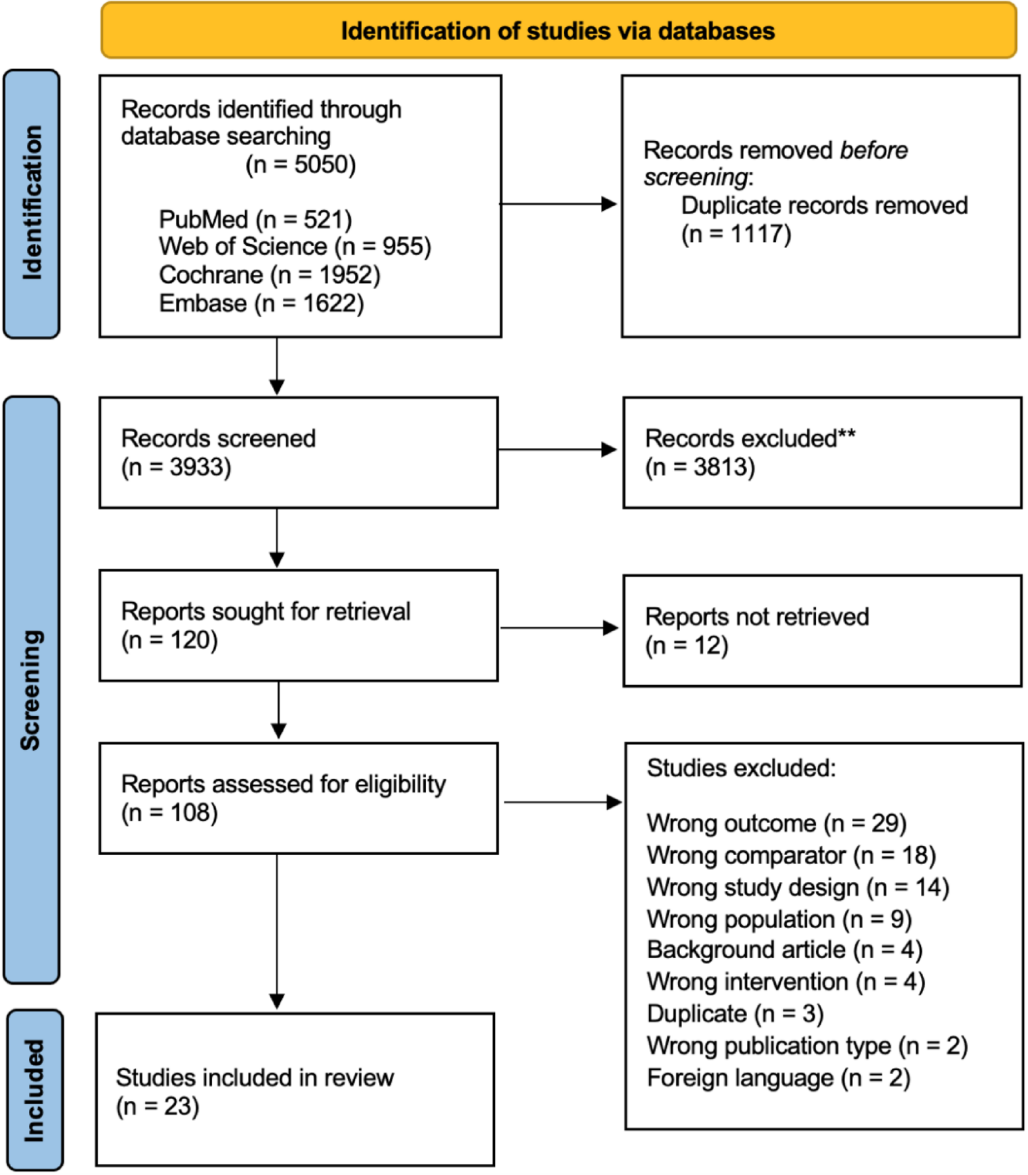
PRISMA Flow Diagram of Study Selection Process

A total of 5050 articles were identified and imported into Rayyan.ai from different databases (521 from PubMed, 955 from Web of Science, 1952 from Cochrane, and 1622 from Embase), with 1117 duplicates excluded. After the removal of duplicates, 3933 articles were screened by four independent reviewers (CG, VM, OQ, PC), based on title and abstract, according to PICOS criteria, leaving 120 reports for retrieval. Of these, 12 were not found, and 108 full-text articles were assessed for eligibility. The full-text screening was independently conducted by four reviewers, with discrepancies resolved through collaborative discussion. Twenty-three studies were included, with eight [3, 4, 17, 23, 30, 34, 39]; Melo Filho et al. 2022) further subdivided due to multiple intervention arms, resulting in a total of 30 studies. In cases of 3-arm RCTs, the two intervention groups were compared to the shared control group, dividing the number of participants of the latter in two, according to the Cochrane handbook recommendations. The results of the search and selection process in the PRISMA flow diagram (Fig. 1).

The characteristics of the included studies are reported in the Supplementary Material Table 3.

### Risk of bias

**Fig. 2:**
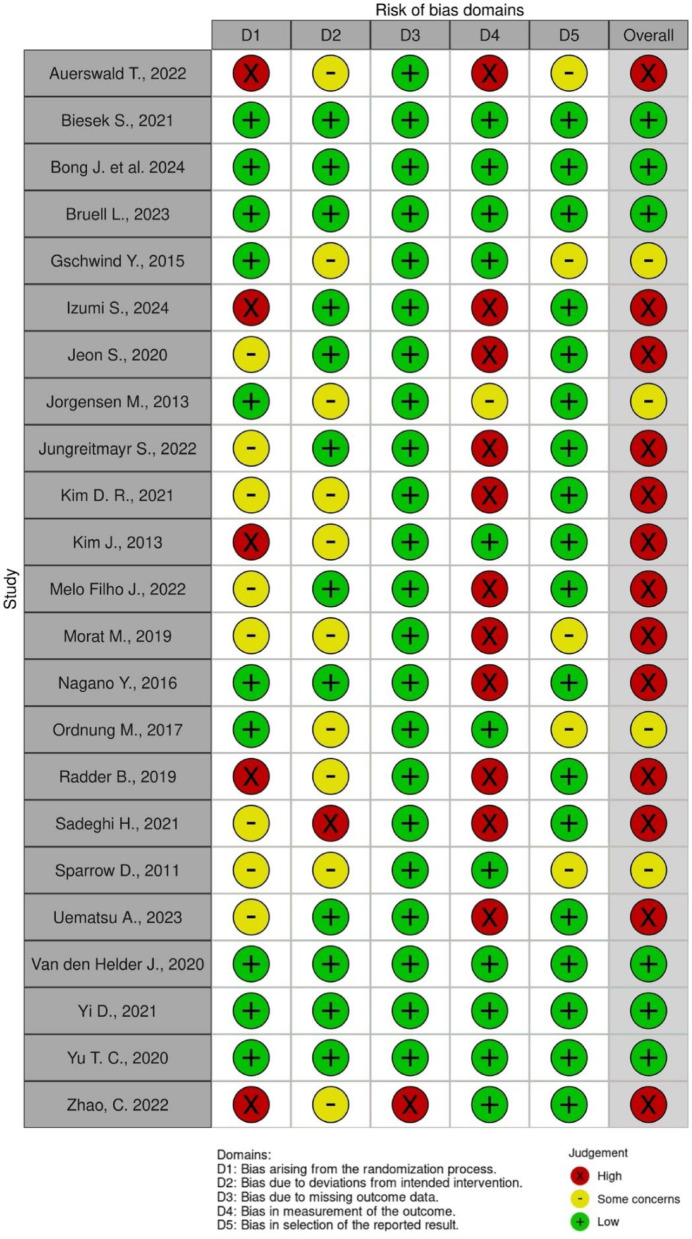
Risk of Bias assessment for each study and overall Risk of Bias by domain

Among the 23 studies, the measurement of the outcome (D4) was identified as a major source of bias, with 11 studies classified as having a high risk of bias in this domain. This was mainly due to common issues, such as incomplete reporting of blinding in measurement procedures. Regarding the randomisation process (D1), five studies were classified as having a high risk of bias, and eight studies raised some concerns. These issues were attributed to inadequate randomisation methods or incomplete reporting of the allocation process. However, 18 studies demonstrated a low risk of bias in the domains related to the selection of reported results (D5). In addition, for bias due to missing outcome data (D3), the risk was low except one [41]. Overall, 13 studies were judged to have a high overall risk of bias, 4 studies exhibited some concerns, and 6 studies had a low risk across all domains. The results of the risk of bias are presented in Fig. 2.

### Meta-analysis

**Fig. 3:**
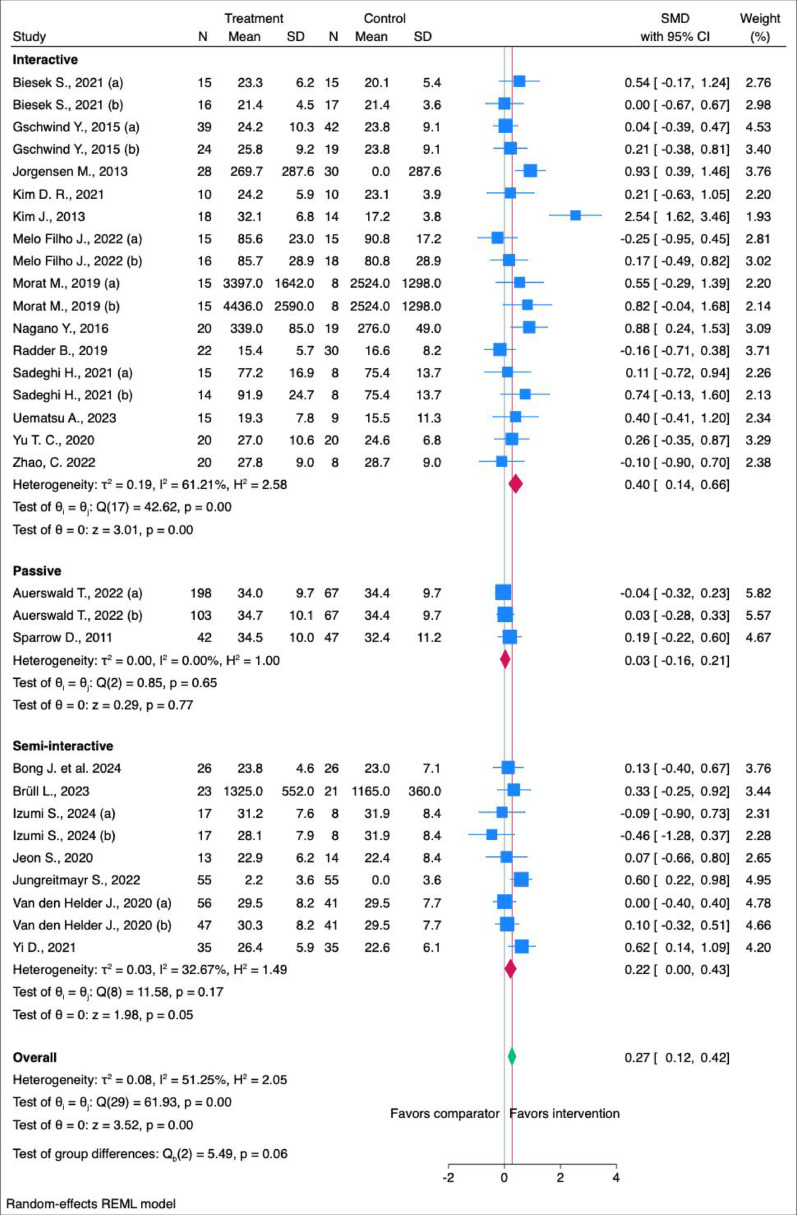
Meta-analysis of the overall, and by type of interventions effects of technology-based PA on MMF. N = Number of participants, SD = Standard Deviation, CI = Confidence Interval, SMD = Standardised Mean Difference

Digital interventions had a statistically significant but small effect on improving muscle mechanical function (SMD: 0.27 [95% CI: 0.12; 0.42] (Fig. 3). Subgroup analysis showed that interactive interventions (*n* = 18) and semi-interactive interventions (*n* = 9) had a statistically significant moderate and small effect, respectively (SMD: 0.40 [95% CI: 0.14; 0.66], 0.22 [95% CI: 0.00; 0.43], while passive interventions (*n* = 3) showed no statistically significant impact (SMD: 0.03 [95% CI: -0.16; 0.21]) (Fig. 3). The findings from one study (Ordnung et al. 2017) were summarized narratively due to the lack of data forinclusion in our meta-analysis. They showed that exergame training significantly improved hand fine motor skills but not sensorimotor and cognitive skills (Ordnung et al. 2017).

**Fig. 4:**
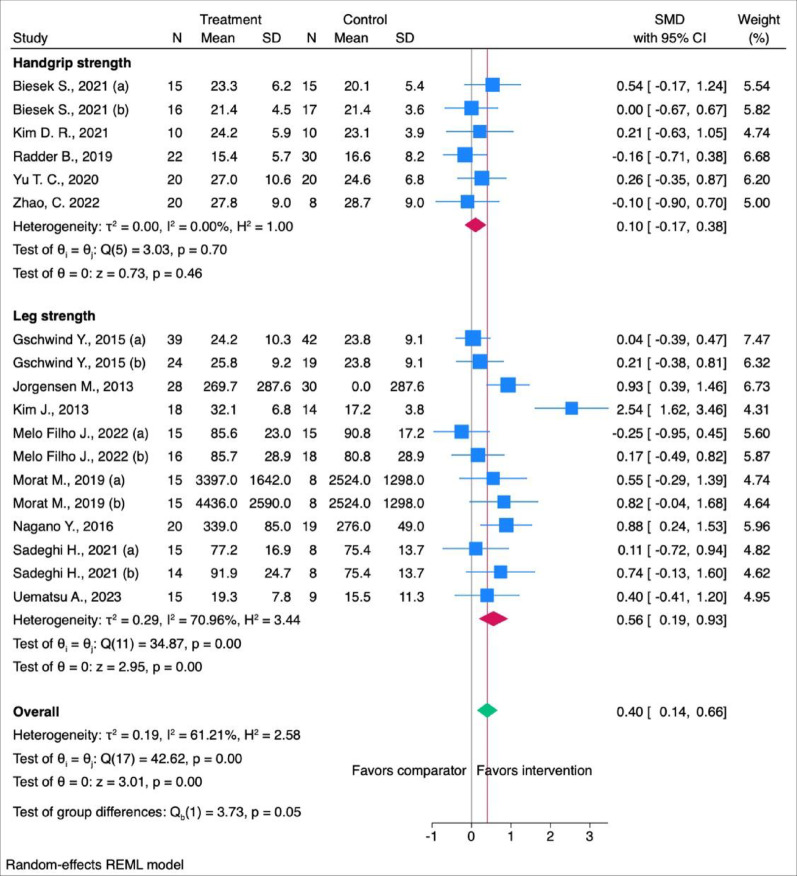
Subgroup analyses for the effects of interactive exercise interventions on MMF for HS and LS. N = Number of participants, SD = Standard Deviation, CI = Confidence Interval, SMD = Standardised Mean Difference

Since the meta-analysis revealed a greater effect of interactive interventions on MMF, the subsequent two sub-analyses will focus exclusively on these 18 studies. For sub-analyses that include all 30 studies, please refer to the supplementary material Fig. 6 and Fig. 7. Only LS showed a statistically significant moderate effect on improving muscle mechanical function (SMD: 0.56 [95% CI: 0.19; 0.93]) (Fig. 4) when muscle mechanical function was stratified by upper and lower body strength (handgrip strength (HS, *n* = 6)) and leg strength (LS, *n* = 12).

**Fig. 5:**
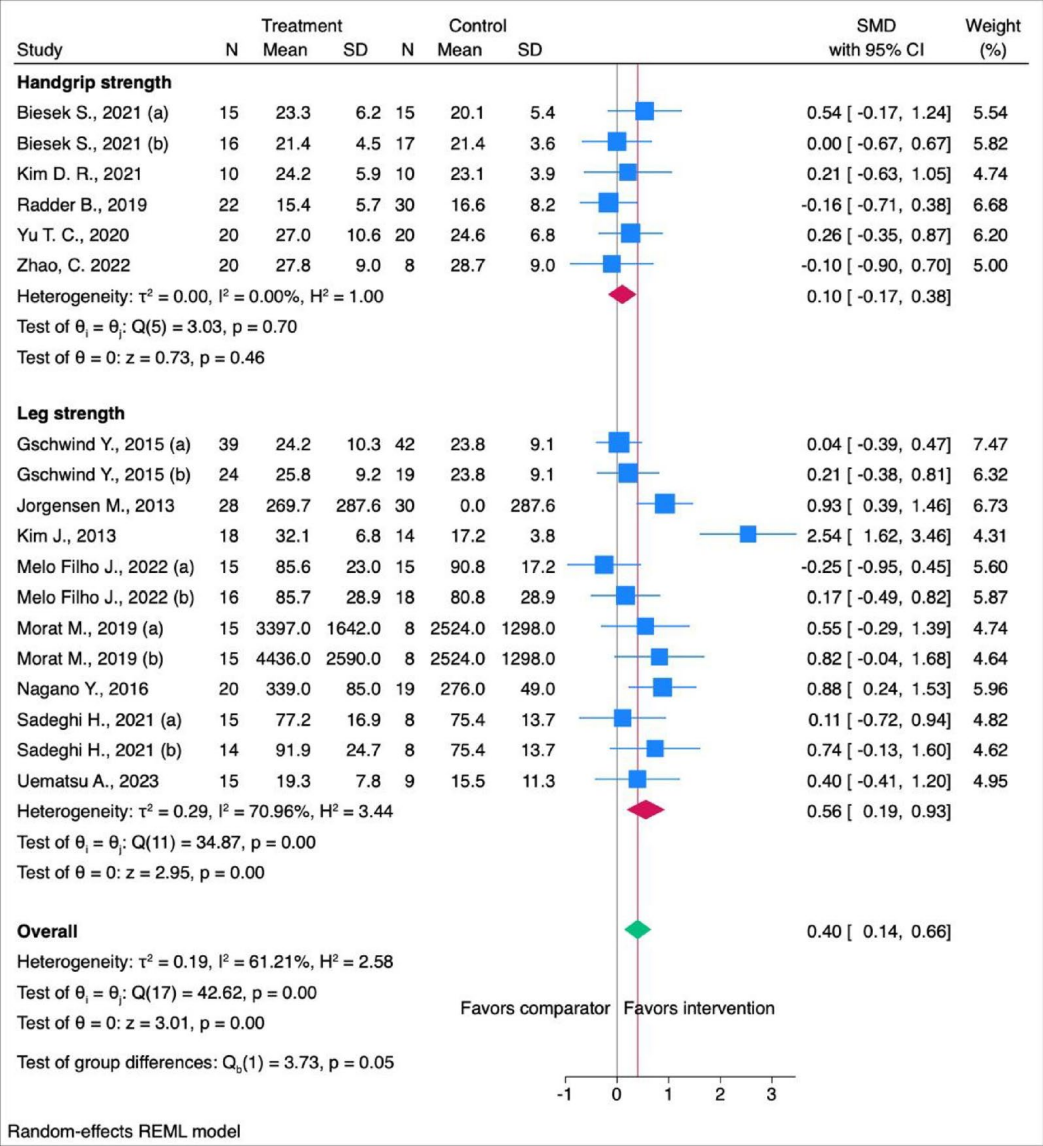
Subgroup analyses for the effects of intensity of the interventions on MMF divided into not supervised and supervised interventions. N = Number of participants, SD = Standard Deviation, CI = Confidence Interval, SMD = Standardised Mean Difference

When MMF was stratified by supervised (*n* = 12) or unsupervised format (*n* = 6), only supervised ones showed a statistically significant moderate effect on MMF (SMD: 0.43 [95% CI: 0.20; 0.67]) (Fig. 5).

### Meta-regression analyses

The meta-regression analyses investigating the impact of participant, interventions, and trial characteristics (age, sex, TUG scores, attendance, number of sessions, and weekly exercise minutes) did not affect the overall results (Supplementary Material Figs. 8–13).

### Certainty of evidence

The overall certainty for HS and LS was downgraded to low for both outcomes due to the ‘high’ or ‘some concern’ judgments (based on the RoB) for most studies and the indirectness of the population (i.e., as few studies included the generalisability of the findings to the entire population of interest that is, older adults is uncertain). We did not deem it appropriate to downgrade for inconsistency as the heterogeneity of the findings could be partially explained by the sub-group analyses on type of supervision and outcome measure (Table 2).

**Table 2:** Assessment of certainty (GRADE) for the primary outcome MMF, divided into HS and LS

Certainty assessment					*N*		Effect	Certainty
S	RoB	Inconsis-tency	Indirect-ness	Impreci- sion	IG	CG	95% CI	
**Handgrip strength**								
16	Serious^a^	Not serious	Not serious	Serious^b^	615	407	SMD 0.07 SD higher (0.06 lower to 0.19 higher)	⨁⨁◯◯ Low^a, b^
**Leg strength**								
15	Very serious^a^	Not serious	Not serious	Very serious^b^	354	321	SMD 0.5 SD higher (0.23 higher to 0.78 higher)	⨁⨁◯◯ Low^a, b^

S = number of studies, RoB = risk of bias, IG = intervention group, CG = control group, CI = confidence interval. The letters represent the following categories:

a = Downgraded one level due to risk of bias

b = Downgraded one level due to indirectness of the population

## Discussion

The main findings of this systematic review and meta-analysis indicate that digital exercise interventions resulted in small but statistically significant improvements in muscle mechanical function among community-dwelling older adults (Fig. 2). Importantly, our secondary analysis revealed that interventions using interactive and semi-interactive digital technology had a statistically significant moderate and small effect, respectively, on MMF, whereas passive interventions had no effect. Although fewer studies used passive interventions, it may be speculated that interactive technologies, such as full-body motion-based gaming consoles, are more effective in enhancing MMF compared to less engaging interventions, such as devices targeting specific muscle groups. Potential explanations may include increased neuromuscular engagement, enhanced motor learning through feedback, or improved adherence via gamification elements.

This is an important finding, as the interactive component of the digital interventions appears to improve adherence through gamification and increased emotional engagement both critical factors for sustaining adherence and potentially the effectiveness of these interventions [22].

Moreover, gaming consoles engage the entire body, targeting multiple muscle groups simultaneously and incorporating gamification elements to enhance user engagement. Notably, exergames designed with numerous interactive elements and delivered through consoles have been shown to improve cognitive function, psychological outcomes, and physical performance in older adults [9]. Finally, although adherence was not consistently reported in the studies included in this meta-analysis, it was earlier highlighted that this mode of exercise delivery had very high adherence (88%). This is likely due to the engaging nature of gaming consoles compared to the traditional exercise programs [24]. Indeed, this was supported by the findings of a previous study [38] reporting a higher adherence rate (91.3%) in the technology-based programs compared to the traditional programs (83.6%), possibly due to a higher level of enjoyment. Another study [28] highlighted high levels of engagement and a positive attitude toward exergame participation.

The secondary analysis regarding the anatomical locations of the assessment indicated that digital interventions improved strength in the lower limbs but not in the upper limbs (Fig. 4). Regardless, it should be noted that (i) fewer studies assessed upper limb compared to lower limb strength (Fig. 4), with (ii) most of the digital-based interventions focused on lower body exercises rather than upper body exercises. The limited number of studies may also partially account for the absence of significant improvement observed in upper limb strength, as meta-analyses based on fewer studies often yield less precise and potentially inconclusive estimates. Additionally [31], emphasises that programs targeting the lower limbs are more effective in counteracting the decline in muscle strength compared to those focusing on the upper limbs.

The supervised versus unsupervised delivery of the interventions is also an essential component of the study design. The studies performed with the supervision of expert trainers during sessions (*n* = 2) showed significant positive effects on muscle mechanical function compared to non-supervised interventions (*n* = 6). This may be partially attributed to the benefits of providing clear instructions on the correct execution of the exercises, which contributes to the perception of safety, as earlier reported [11]. This is particularly crucial for older individuals who may have pre-existing health conditions or limited physical capabilities [15]. Moreover, motivation to adhere to the exercise intervention may be enhanced through social interaction between the operator and the participant. Implementing a supervised program, either routinely or occasionally, may therefore be an important aspect to consider when designing digital interventions for older adults. Regardless, results regarding the role of supervision should be interpreted cautiously as the number of supervised studies were twice as many (*n* = 12) compared to non-supervised studies (*n* = 6). Reporting the specific elements of an exercise intervention such as intensity, modality and time (FITT principle) is an essential aspect for critically interpreting the results of the intervention and understanding key issues such as dose-response. It was earlier reported that longer and more frequent exercise sessions (e.g., 50–60 min, 2–3 times per week) lead to greater improvements [7].

In general, nearly all studies included in this review reported (i) duration of the single exercise sessions, (ii) weekly frequency, and (iii) total duration of the digital interventions, but they lacked information on intensity, making the comparisons among interventions very difficult.

## Limitations

This systematic review has several limitations. First, the analysis was limited to interventions focusing exclusively on physical activity delivered through digital technologies, excluding those combining digital approaches with other forms of physical activity. This exclusion limits the generalizability of the findings, particularly concerning the effectiveness of multimodal interventions that integrate diverse activity methods.

Second, the study population was limited to “healthy” older adults, excluding individuals with chronic conditions or specific pathologies. As a result, the findings may not apply to more clinically diverse or vulnerable populations, such as those with multiple chronic conditions or significant functional impairments.

Although the literature search was conducted up to March 2024, we acknowledge that new evidence may emerge in this rapidly evolving field. Therefore, we will consider updating the evidence base within the next five years, should relevant studies become available.

Another important limitation of this review is the overall high risk of bias across the included studies: among the 23 studies, only 6 were judged as having a low risk of bias, 4 had some concerns, and 13 were classified as having a high risk of bias. The high prevalence of risk of bias, particularly related to outcome measurement and randomisation procedures, may have led to either an overestimation or underestimation of the effect sizes in the individual studies. Consequently, the certainty was downgraded due to the concerns about risk of bias.

## Conclusion

This systematic review and meta-analysis found that digitally delivered physical exercise interventions have a small but statistically significant effect on muscle strength in older people. Interactive and semi-interactive formats appeared more effective than non-interactive intervention designs, and supervised interventions generally yielded better muscle function outcomes than unsupervised ones.

However, the absence of detailed reporting on key exercise variables ( particularly intensity, limited comparability across studies and reduced the confidence in the findings. Furthermore, the overall certainty of the evidence was low due to the small number of the included studies, high risk of bias and insufficient reporting of intervention and comparator characteristics. Thereby these findings should be cautiously interpreted and further high-quality randomised controlled trials are warranted to strengthen the evidence of our findings.

## Supplementary Information

Below is the link to the electronic supplementary material.

Supplementary Material 1

## Data Availability

No datasets were generated or analysed during the current study.

## Abbreviations

MMF: Muscle Mechanical Function
HS: Handgrip Strength
LS: Leg Strength
TC: Technology Component
ToI: Type of Intervention
NC: Number of Components
RPE: Rate Perceived Exertion
BMI: Body Mass Index
TUG: Timed up-and-go test
PA: Physical Activity
IG: Intervention Group
CG: Control Group
