# Supplementary material for: Effects of digital physical activity interventions on muscle mechanical function in community-dwelling older adults: a systematic review and meta-analysis
Source: Eur Rev Aging Phys Act. 2025 Sep 2;22:14. doi: 10.1186/s11556-025-00380-z (PMC12403258; doi:10.1186/s11556-025-00380-z)
Supplement: Supplementary file 1 — Supplementary Material 1 [file 11556_2025_380_MOESM1_ESM.zip › Materiale supplementare 3/Supplementary Material Figure 6 .docx]

### **Supplementary Material**


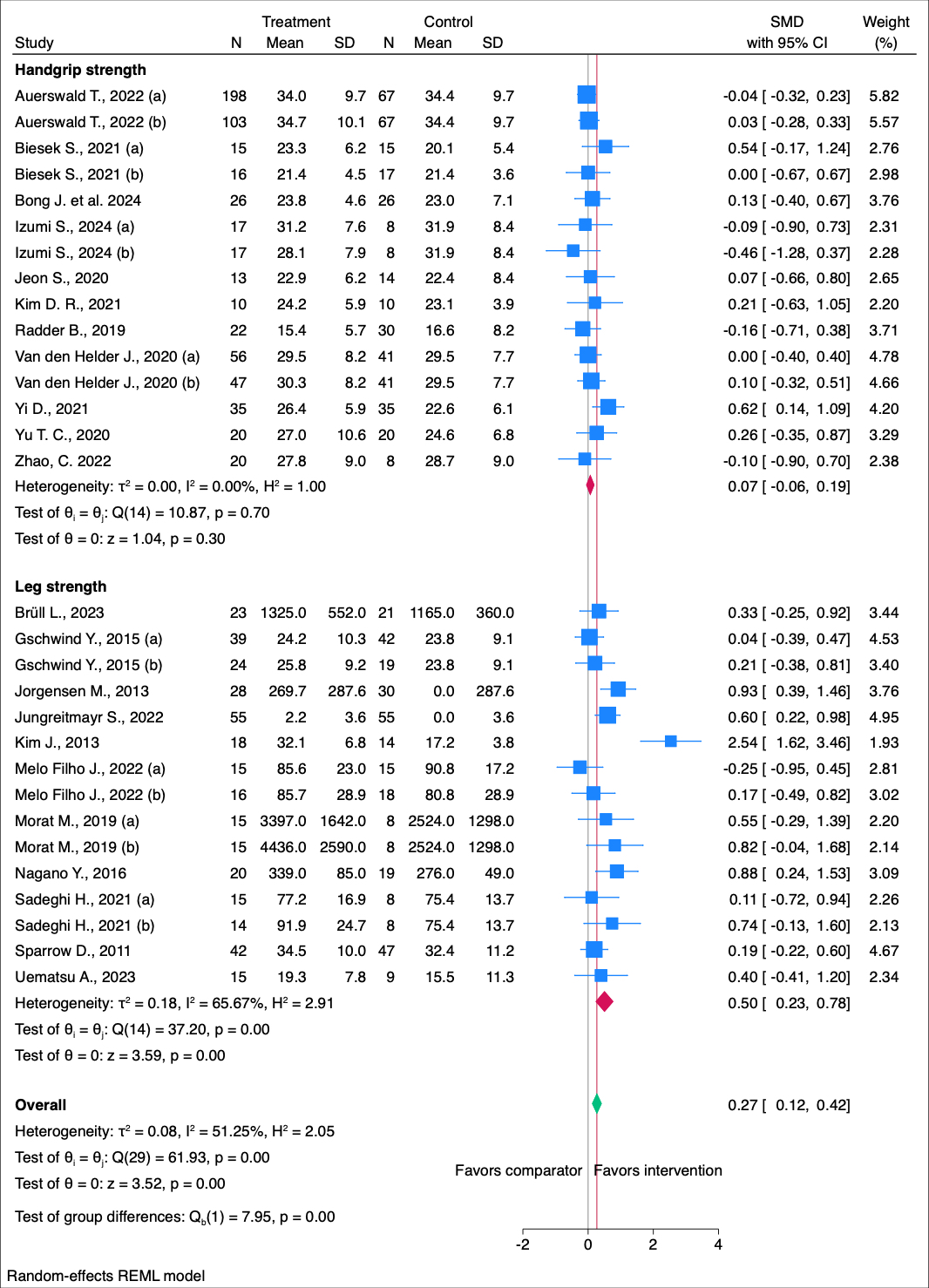


Figure 6: Sub-analysis of the Handgrip Strength and Leg Strength for all studies.
