# Supplementary material for: Effects of digital physical activity interventions on muscle mechanical function in community-dwelling older adults: a systematic review and meta-analysis
Source: Eur Rev Aging Phys Act. 2025 Sep 2;22:14. doi: 10.1186/s11556-025-00380-z (PMC12403258; doi:10.1186/s11556-025-00380-z)
Supplement: Supplementary file 1 — Supplementary Material 1 [file 11556_2025_380_MOESM1_ESM.zip › Materiale supplementare 3/Supplementary material meta regression Figure 12.docx]

### **Supplementary Material**

The effect sizes for MMF and session duration (in minutes) (n = 18) were used to conduct a meta-regression and visualise the results in a bubble plot (Figure 10). β was 0.001 [-0.015; 0.017], p = 0.902, indicating that an increase of 1 effect size in % of attendance gives a non-significant increase of 0.001 effect sizes in MMF. The R^2^ was 0.00%, indicating a high heterogeneity between studies.


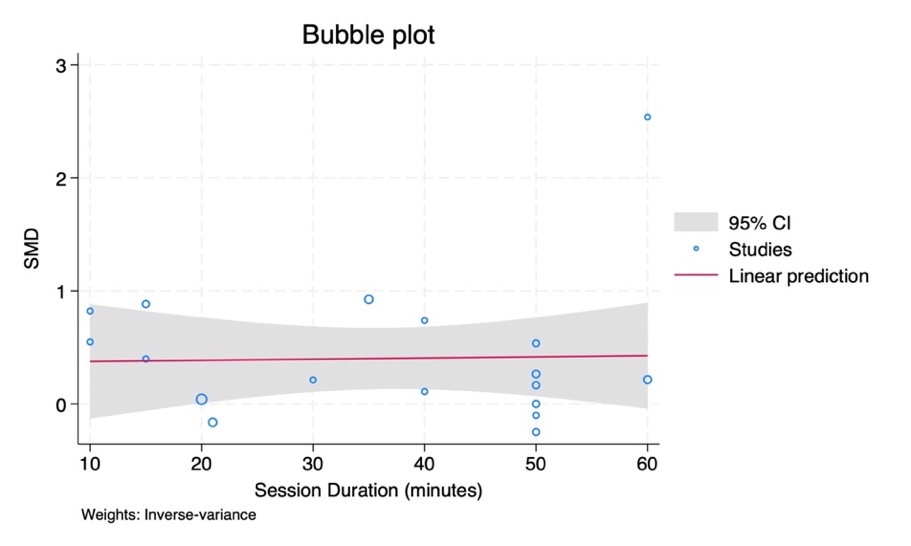


Figure 10: Meta-regression of the concomitant changes between MMF and session duration in minutes.
