# Supplementary material for: Effects of digital physical activity interventions on muscle mechanical function in community-dwelling older adults: a systematic review and meta-analysis
Source: Eur Rev Aging Phys Act. 2025 Sep 2;22:14. doi: 10.1186/s11556-025-00380-z (PMC12403258; doi:10.1186/s11556-025-00380-z)
Supplement: Supplementary file 1 — Supplementary Material 1 [file 11556_2025_380_MOESM1_ESM.zip › Materiale supplementare 3/Supplementary material meta regression Table 4.docx]

### **Supplementary Material**

Table 4: Main results of meta-regressions interactive interventions.

| Meta-regressions (interactive) | | | | |
| --- | --- | --- | --- | --- |
| Outcomes | **Nr. of studies** | **β (95% CI)** | **P-value** | **R^2^ (%)** |
| MMF & Mean Age | 18 | -0.023 [-0.081; 0.034] | 0.432 | 0.00 |
| MMF & Gender | 18 | -0.002 [-0.011; 0.006] | 0.549 | 0.00 |
| MMF TUGs | 11 | -0.051 [-0.17; 0.066] | 0.393 | 1.89 |
| MMF & Attendance | 16 | -0.000 [-0.046; 0.046] | 0.988 | 0.00 |
| MMF & Sess. Durat. | 18 | 0.001 [-0.015; 0.017] | 0.902 | 0.00 |
| MMF & Min/Week | 18 | 0.000 [-0.006; 0.006] | 0.952 | 0.00 |

N = number of participants, β = slope coefficient, CI = confidence interval, p-value = significance of ≤0.05, R^2^ = statistical heterogeneity. MMF = muscle mechanical function. Gender = % of females, TUGs = timed up and go in seconds, attendance = in %, sess. Durat. = session duration.
