# Supplementary material for: Effects of digital physical activity interventions on muscle mechanical function in community-dwelling older adults: a systematic review and meta-analysis
Source: Eur Rev Aging Phys Act. 2025 Sep 2;22:14. doi: 10.1186/s11556-025-00380-z (PMC12403258; doi:10.1186/s11556-025-00380-z)
Supplement: Supplementary file 1 — Supplementary Material 1 [file 11556_2025_380_MOESM1_ESM.zip › Materiale supplementare 3/Supplementary Material Table 2 Search Matrix.docx]

**Supplementary Material**

Table 2: Full search matrix for PubMed.

| **Concept** | **Search query** |
| --- | --- |
| **Participants: Older Adults (60+)** | "Aged"[Mesh] OR “Aged, 80 and over"[Mesh] OR "Aging"[Mesh] OR "Frail Elderly"[Mesh] OR “Older adult*”[tw] OR senior*[tw] OR elderly*[tw] OR “old people”[tw] OR “old person”[tw] OR “old persons”[tw] or “old individuals”[tw] OR “old adults”[tw] OR “aged adult*”[tw] OR “frail elderly*”[tw] OR “frail old*”[tw] OR “centenarian*”[tw] OR “nonagenarian*”[tw] OR  “octogenarian*”[tw] OR “elder*”[tw] OR “aging”[tw] OR “ageing”[tw] OR "older patient*"[tw] OR "older women"[tw] OR "older men"[tw] OR "older individual*"[tw] OR "older population"[tw] OR "older age*"[tw] OR “geriatric*”[tw] OR "senior citizen*"[tw] OR “seniors”[tw] |
| **Intervention: (Technology-based) Exercise** | "Exercise"[Mesh] OR "Exercise Therapy"[Mesh] OR "Sports"[Mesh] OR "Exercise Test"[Mesh] OR “exercis*”[tw] OR “recreation"[tw] OR "Physical activit*"[tw] OR "physical training"[tw] OR “sport*”[tw] OR "Circuit-Based Exercise"[Mesh] OR "physical activity"[tw] OR “walking”[tw] OR “yoga”[tw] OR "tai-chi"[tw] OR "balance training"[tw] OR "strength training"[tw] OR "functional training"[tw] OR "resistance training"[tw] OR "aerobic training"[tw] OR "multi-component training"[tw] OR "circuit-training"[tw] OR  "Resistance Training"[Mesh] OR "Endurance Training"[Mesh] OR “Exercise*”[tw] OR “physical activity*”[tw] OR “resistance training*”[tw] OR “strength training*”[tw] OR “weightlifting*”[tw] OR “weight exercise*”[tw] OR “power training*”[tw] OR “strengthening exercise*”[tw] OR “resistance exercise*”[tw] OR “resistive exercise*”[tw] OR “resistive training*”[tw] OR “HIIT”[tw] OR “High intensity interval training”[tw] OR “SIT”[tw] OR “Sprint interval training”[tw] OR “home-based”[tw] OR “body weight exercise”[tw] OR “exercise therapy”[tw] OR “calisthenics”[tw] OR “olympic lifting”[tw] OR “Powerlifting”[tw] OR “Blood flow restriction”[tw] OR “BFR”[tw] OR “exercise”[tiab] OR "muscle activity"[tiab] OR “swim*”[tiab] OR “gym”[tiab] OR “gymn*”[tiab] OR “walk*”[tiab] OR “danc*”[tiab] OR “jog”[tiab] OR “jogg*”[tiab] OR “run”[tiab] OR “runn*”[tiab] OR” “sprint*”[tiab] OR “cycl*”[tiab] OR “bicycl*”[tiab] OR “biking”[tiab] OR “hiking”[tiab] OR “basket”ball”[tiab] OR “baseball”[tiab] OR “boxi*”[tiab] OR “box”[tiab] OR “boxe*”[tiab] OR “football”[tiab] OR “judo”[tiab] OR “soccer”[tiab] OR “golf*”[tiab] OR “hockey”[tiab] OR “rugby”[tiab] OR "martial arts"[tiab] OR “skating”[tiab] OR “skiing”[tiab] OR “volleyball”[tiab] OR “wrestl*”[tiab] OR "weight lift*"[tiab] OR cricket[tiab] OR "tai ji"[tiab] OR "tai chi"[tiab] OR yoga[tiab] OR "qi gong"[tiab] OR sport*[tiab] OR "physical training"[tiab] OR "strength training"[tiab] OR "weight training"[tiab] OR "resistance training"[tiab] OR "high-intensity interval training"[tiab] OR "balance training"[tiab] OR "aerobic training"[tiab] OR "cardiovascular training"[tiab] OR "cardiorespiratory training"[tiab] OR "blood flow restriction training"[tiab] OR "occlusion training"[tiab] OR "eccentric training"[tiab] OR "concentric training"[tiab] OR "isokinetic training"[tiab] OR "hypertrophy training"[tiab] OR "power training"[tiab] OR “plyometric”[tiab] OR “kaatsu”[tiab] OR "circuit training"[tiab] OR "stability training"[tiab] OR "body training"[tiab] OR "mind-body training"[tiab] OR "anaerobic training"[tiab] OR "endurance training"[tiab] OR "muscle training"[tiab] OR "active video gam*"[tiab] OR “Wii”[tiab] OR “Kinect”[tiab] OR “pilates”[tiab] OR “feldenkrais”[tiab] OR "motor activity"[tiab] OR "locomotor activity"[tiab] O OR "physical rehabilitation"[tiab] OR "Wingate test"[tiab] AND "Telemedicine"[Mesh] OR "Internet-Based Intervention"[Mesh] OR “e-health”[tw] OR “mhealth”[tw] OR "technology assisted"[tw] OR “web-based”[tw] OR “online”[tw] OR “remote*”[tw] OR “exergames”[tw] OR "virtual reality"[tw] OR "smart technology"[tw] OR “wearables”[tw] OR “Telerehabilitation”[tw] OR “Web*”[tw] OR “Mhealth”[tw] OR “m-health”[tw] OR “technolog*”[tw] OR “gamification”[tw] OR “gamif*”[tw] OR “augmented reality”[tw] OR “AR”[tw] OR “Tele-rehab”[tw] OR “Tele rehab*”[tw] OR “Tele-health”[tw] OR “Tele health”[tw] OR “Sensor-based”[tw] OR “Sensor”[tw] OR “Virtual training”[tw] OR “Virtual exercise”[tw] |
| **Outcome: Muscle Mechanical Function** | "Muscle Strength"[Mesh] OR “mechanical muscle function”[tw] OR “muscle mechanical function”[tw] OR “muscle force”[tw] OR “muscle power”[tw] OR “explosive force”[tw] OR “explosive strength”[tw] OR “rapid force”[tw] OR “muscle strength”[tw] OR “rate of force development*”[tw] OR “RFD”[tw] OR “maximal voluntary contraction*”[tw] OR “force steadiness”[tw] OR “force control”[tw] OR “force fluctuation”[tw] OR “stiffness”[tw] OR -“impulse”[tw] OR “rapid muscle force”[tw] OR “muscle stiffness”[tw] OR “muscle-tendon complex”[tw] OR “force-velocity”[tw] OR “power-velocity”[tw] OR “pennation angle”[tw] |
