# Supplementary material for: Effects of digital physical activity interventions on muscle mechanical function in community-dwelling older adults: a systematic review and meta-analysis
Source: Eur Rev Aging Phys Act. 2025 Sep 2;22:14. doi: 10.1186/s11556-025-00380-z (PMC12403258; doi:10.1186/s11556-025-00380-z)
Supplement: Supplementary file 1 — Supplementary Material 1 [file 11556_2025_380_MOESM1_ESM.zip › Materiale supplementare 3/Supplementary Material Table 3 Study Characteristics.docx]

**Supplementary Material**

Table 3: Study characteristics of the studies included.

| Author, year | Country | Age (years, mean) | Sample Size | Gender (female%) | BMI (kg/m^2^, mean) | Technology Type of Intervention | Technology Component | PA Intervention Type | TTS  (SPW x TW) | Session duration (min) | Comparator | Outcome |
| --- | --- | --- | --- | --- | --- | --- | --- | --- | --- | --- | --- | --- |
| Auerswald T., 2022 (a) | D | 69.19 | 332 | 76.5 | 27.55 | P | D | RT + B + E + F | 84 (7x12) | 10 | WL | HS |
| Auerswald T., 2022 (b) | D | 70.15 | 237 | 54.8 | 27.50 | P | D | RT+ B + E + F | 85 (7x12) | 10 | WL | HS |
| Biesek S., 2021 (a) | BRA | 70.8 | 30 | 100 | 28.6 | **IN** | C | RT + N | 24 (2 x 12) | 50 | US | HS |
| Biesek S., 2021 (b) | BRA | 72.4 | 33 | 100 | 29.2 | **IN** | C | RT + N | 24 (2 x 12) | 50 | SU | HS |
| Bong J. et al. 2024 | MAS | 66.54 | 52 | 84.62 | N/A | SI | V | RT + B + E + S | 48 (2 x 24) | 75-90 | US + 1 EDS | HS |
| Bruell L., 2023 | D | 75.45 | 44 | 70 | 24.7 | SI | D + V | B | 18 (3 x 6) | 24 | US | LS |
| Gschwind Y., 2015 (a) | AUS | 81.3 | 81 | 67.14 | 27.11 | **IN** | C | S | 48 (3 x 16) | 20 | US + MED | LS |
| Gschwind Y., 2015 (b) | AUS | 80.14 | 43 | 64.7 | 26.60 | **IN** | C | RT + B | 32 (2 x 16) | 60 | US + MED | LS |
| Izumi S., 2024 (a) | JPN | 70.5 | 25 | 68 | 26.22 | SI | V | RT | 24 (2 x 12) | 60 | US | HS |
| Izumi S., 2024 (b) | JPN | 69.61 | 25 | 68 | 25.94 | SI | V | RT | 24 (2 x 12) | 40 | US | HS |
| Jeon S., 2020 | KOR | 72.74 | 27 | 100 | 27.78 | SI | D | RT + E + F | 60 (5 x 12) | 30 | NOEX | HS |
| Jorgensen M., 2013 | DK | 74.76 | 58 | 69 | 26.14 | **IN** | C | RT + B | 20 (2 x 10) | 35 | SI | LS |
| Jungreitmayr S., 2022 | AUT | 65.3 | 110 | 100 | N/A | SI | A | RT + B | 28 (2 x 14) | 10/20/30 | WL | LS |
| Kim D. R., 2021 | KOR | 66.51 | 20 | 85 | 23.66 | **IN** | V + C | E | 24 (2 x 12) | 30 | US | HS |
| Kim J., 2013 | KOR | 67.37 | 32 | 84.37 | 24.78 | **IN** | C | RT + B | 24 (3 x 8) | 60 | US | LS |
| Melo Filho J., 2022 (a) | BRA | 70.7 | 30 | 100 | 28.9 | **IN** | C | ME | 24 (2 x 12) | 50 | US | LS |
| Melo Filho J., 2022 (b) | BRA | 72.42 | 34 | 100 | 29.4 | **IN** | C | RT + B + N | 25 (2 x 12) | 50 | SU | LS |
| Morat M., 2019 (a) | D | 70.19 | 23 | 52 | 28.09 | **IN** | D | S | 24 (3 x 8) | 10-12 | US | LS |
| Morat M., 2019 (b) | D | 68.75 | 23 | 48 | 26.79 | **IN** | D | S | 24 (3 x 8) | 10-12 | US | LS |
| Nagano Y., 2016 | JPN | 72 | 39 | 31 | 23.51 | **IN** | D | S | 24 (2 x 12) | 15 | US | LS |
| Ordnung M., 2017 | D | 69.21 | 29 | 51.72 | 25.97 | **IN** | C | ME | 12 (2 x 6) | 60 | US | HS |
| Radder B., 2019 | NED | 72 | 52 | 69.23 | N/A | **IN** | D | RT + C | 28 (7 x 4) | 21 | US | HS |
| Sadeghi H., 2021 (a) | MAS | 73.44 | 23 | 0 | 24.34 | **IN** | C | B | 24 (3 x 8) | 40 | US | LS |
| Sadeghi H., 2021 (b) | MAS | 71.12 | 22 | 0 | 24.91 | **IN** | C | B | 24 (3 x 8) | 40 | US | LS |
| Sparrow D., 2011 | USA | 71.1 | 89 | 31.07 | 28.8 | P | P | RT | 156 (3 x 52) | 60 | ED | LS |
| Uematsu A., 2023 | JPN | 75.45 | 24 | 44 | N/A | **IN** | C | B | 16 (2 x 8) | 15 | US | LS |
| Van den Helder J., 2020 (a) | NED | 72.51 | 97 | 72 | 25.46 | SI | A | RT + E + B + F + FT | 52 (2 x 26) | N/A | US | HS |
| Van den Helder J., 2020 (b) | NED | 71.61 | 88 | 72 | 26.47 | SI | A | RT + E + B + F + FT | 52 (2 x 26) | N/A | US | HS |
| Yi D., 2021 | KOR | 76.71 | 70 | 82 | 24.43 | SI | V | RT + B | 16 (2 x 8) | 40 | US | HS |
| Yu T. C., 2020 | TW | 64 | 40 | 80 | 23.27 | **IN** | C | ME | 30 (3 x 10) | 50 | US | HS |
| Zhao, C. 2022 | CHN | 65.68 | 28 | 63.15 | 23.55 | **IN** | C | ME | 36 (3 x 12) | 50-55 | US | HS |

*TTS = total training sessions, SPW = sessions per week, TW = total weeks. P = passive, IN = interactive, SI = semi-interactive. D = device, C = console, V = video, A = app, P = phone. RT = resistance training, B = balance, C = coordination, N = neuromuscular, S = stepping, E = endurance, ME = mixed exergames, F = flexibility, FT = functional training. WL = wait list, US = usual care, SU = supplement, SI = shoe insoles, M = minimal, ED = education, NOEX = no exercise, S = session; HS = handgrip strength, LS = leg strength*
