# Supplementary material for: Effects of digital physical activity interventions on muscle mechanical function in community-dwelling older adults: a systematic review and meta-analysis
Source: Eur Rev Aging Phys Act. 2025 Sep 2;22:14. doi: 10.1186/s11556-025-00380-z (PMC12403258; doi:10.1186/s11556-025-00380-z)
Supplement: Supplementary file 1 — Supplementary Material 1 [file 11556_2025_380_MOESM1_ESM.zip › Materiale supplementare 3/Supplementary material meta regression Figure 9.docx]

### **Supplementary Material**

The effect sizes for MMF and gender (percentage of females) (n = 18) were used to conduct a meta-regression and visualise the results in a bubble plot (Figure 7). β was -0.003 [-0.012; -0.006], p = 0.549, indicating that an increase of 1 effect size in % of females gives a non-significant decrease of 0.003 effect sizes in MMF. The R^2^ was 0.00%, indicating a high heterogeneity between studies.


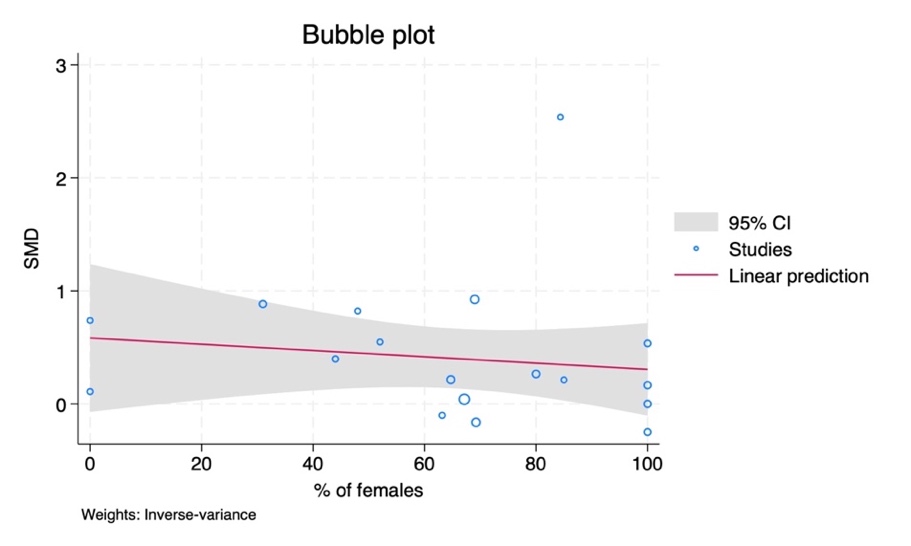


Figure 7: Meta-regression of the concomitant changes between MMF and % of females.
